# Antioxidant Activity as an Indicator of the Efficiency of Plant Extract-Mediated Synthesis of Zinc Oxide Nanoparticles

**DOI:** 10.3390/antiox12040784

**Published:** 2023-03-23

**Authors:** Joelis Vera, Wence Herrera, Edward Hermosilla, Marcela Díaz, Javiera Parada, Amedea B. Seabra, Gonzalo Tortella, Héctor Pesenti, Gustavo Ciudad, Olga Rubilar

**Affiliations:** 1 Doctorate in Sciences Engineering with Specialization in Bioprocess, Faculty of Engendering and Science, Universidad de La Frontera, Temuco 4811230, Chile; j.vera12@ufromail.cl; 2 Doctoral Program in Sciences of Natural Resources, Faculty of Engendering and Science, Universidad de La Frontera, Temuco 4811230, Chile; w.herrera02@ufromail.cl; 3 Biotechnological Research Center Applied to the Environment (CIBAMA-BIOREN), Faculty of Engendering and Science, Universidad de La Frontera, Temuco 4811230, Chile; edward.hermosilla@ufrontera.cl (E.H.); marcela.diaz@ufrontera.cl (M.D.); javiera.parada@ufrontera.cl (J.P.); gonzalo.tortella@ufrontera.cl (G.T.); 4 Chemical Engineering Department, Faculty of Engendering and Science, Universidad de La Frontera, Temuco 4811230, Chile; gustavo.ciudad@ufrontera.cl; 5 Center for Natural and Human Sciences, Universidade Federal do ABC, Santo André 09210-580, Brazil; amedea.seabra@ufabc.edu.br; 6 Núcleo de Investigación en Bioprocesos y Materiales Avanzados, Facultad de Ingeniería, Universidad Católica de Temuco, Temuco 4810302, Chile; hpesenti@uct.cl; 7 Instituto del Medio Ambiente (IMA), Universidad de La Frontera, Temuco 4811230, Chile

**Keywords:** green synthesis, zinc oxide nanoparticles, antioxidant activity, phenolic compounds, plant extract

## Abstract

The green synthesis of zinc oxide nanoparticles (ZnO NPs) using a diverse range of plant species has been extensively reported. Despite the success achieved by biogenic synthesis, there are problems with the control and prediction of the properties of ZnO NPs, due to phytochemical diversity between plant species. In this sense, the main objective of our work was to investigate the effect of the antioxidant activity (AA) of plant extracts on the physicochemical characteristics of ZnO NPs (production yield, chemical composition, polydispersity index (PDI), surface charge (ζ-potential) and average particle size). In order to accomplish this objective, four plant extract with different antioxidant activities were used: *Galega officinalis*, *Buddleja globosa*, *Eucalyptus globulus*, and *Aristotelia chilensis*. Phytochemical screening, quantitative analysis of phenolic compounds and antioxidant activity determination of the different extracts were carried out. Chemical species such as catechin, malvidin, quercetin, caffeic acid, and ellagic acid were the dominant components, found in the extracts studied. The *A. chilensis* extract showed the highest value of total phenolic compounds (TPC) and AA, followed by *E. globulus*, *B. globosa* and *G. officinalis*. Zetasizer, Fourier-transform infrared (FTIR), X-ray diffraction (XRD), transmission electron microscopy (TEM) and thermogravimetric analysis (TGA) data show that plant extracts with lower AA leads to a decrease in the yield of ZnO NPs and an increase in the amount of residual organic extract that remains on the particles. The latter caused an increase in the average particle size, PDI and ζ-potential as a consequence of agglomeration and particle coarsening. Our result suggest that it is possible to use the AA as an indicator of the potential reducing capacity of plant extracts. In this way it is possible to guarantee the reproducibility of the synthesis process as well as ensure the formation of ZnO NPs with desired characteristics.

## 1. Introduction

Zinc oxide nanoparticles (ZnO NPs) have unique characteristics, including thermal stability, optical properties, nontoxic behavior, low cost and ease of fabrication. These properties make them attractive for used in biomedicine, agriculture, textile, solar cells and cosmetic products [1,2,3].

ZnO NPs are generally synthesized by chemical methods due to their high precision and better control over particle size, shapes, and composition [4]. However, the use of highly concentrated reductants and stabilizing agents in the process can provoke a harmful impact on the environment and human health. In this sense, great emphasis has been placed on the green synthesis of ZnO NPs to minimize toxic effluent production and promote environmental sustainability [5]. Green synthesis uses aqueous extracts of different parts of the plant (roots, stem, leaf, flower, and fruit), cellular biomass, or cell-free extracts (of bacteria, fungi, yeasts, and viruses) for nanoparticle production. Plant-based nanoparticle synthesis is highly preferred over synthesis by microorganisms considering its simplicity, scalability, the rich biodiversity of plant species, and their wide range of phytochemical compounds (e.g., terpenoids, tannins, steroids, saponins, polyphenols, alkaloids, phenolic acids, and proteins) [5,6]. The specific mechanisms of plant-mediated synthesis are still uncertain due to the diverse nature of phytochemical constituents [5]. Until now, phenolic compounds have been suggested as being responsible for the phytosynthesis of nanoparticles [7,8]. The antioxidant activity of phenolic compounds allows them to act as reducing agents and stabilizing agents during the preparation of nanomaterials [9,10,11,12].

Considering that the phenolic compounds in plant extracts play a critical role in nanoparticle formation and growth, selecting the right plant source to prepare the extracts is an important step to obtain ZnO NPs with the desired physicochemical properties. In this context, the following question arises: What are the characteristics that must be taken into consideration when choosing a plant source for the synthesis of nanoparticles? Several researchers have documented that the use of different plant species [13,14], different parts of the plant [15] and different plant extract concentrations [16,17] can affect the morphology and particle size of the obtained ZnO [6,18]. To the best of our knowledge, little has been said about the purity and yield of the final product, which are also important properties that could affect its reactivity, hinder its application in the construction of functionalized materials and limit its scalability.

According to Zeghoud et al. [19] unless the plant extract is devoid of bioactive species, the plant species employed has little impact on the production of nanoparticles. However, the biochemical parameters of plant extracts that can be used as practical and quantitative guide for selecting the better biological agent for the fabrication of ZnO NPs have not yet been defined yet, which is necessary to guarantee the reproducibility of the synthesis process, as well as more control over particles properties.

To date, a large number of plant sources have been reported to be able to promote the synthesis of ZnO NPs [18,19,20]. Leaf extract of *Galega officinalis*, *Buddleja globosa* (matico), *Aristotelia chilensis* (maqui) and *Eucalyptus globulus* were selected for this study on the basis of their known medicinal properties. All of these plants grow abundantly in the central–southern region of Chile. *G. officinalis* and *E. globulus* are species that were introduced in the country. *G. officinalis* is employed as medicine and in forage cultures, the maximum amount of phytochemicals is found in its leaves, which contains alkaloids, glycoside, flavonoids, tannins, hydroxycinnamic acids, saponins, bitters, pectins, essential amino acids and vitamins [21]. The leaves of *E. globulus* are residue from the wood industry, and contain flavonoids, volatile oils, and tannins; due to this, they are used for medicinal purposes, especially against coughs and as an expectorant, but they also have febrifuge, antiseptic, hemostatic, and vermifungal properties [22,23]. *B. globosa* and *A. chilensis* are traditional native Chilean plants. *B. globosa* leaf extract is often used in Mapuche culture for cutaneous wound healing, intestinal and liver problems. It has bioactive compounds such as flavonoids, phenylethanoids, sterols, phenolic fatty acid esters and terpenes [24]. Leaves of *A. chilensis* are a waste product of the maqui berry industry. Leaf infusion are used as a natural muscle relaxant or as an analgesic, due to being rich in phenols, a wide variety of anthocyanins, and flavonoids [25]. The bioactivity of all of these plants makes them potential candidates for the biosynthesis of ZnO NPs. 

*E. globulus* leaf extracts have been successfully used for the production of ZnO NPs with spherical morphology and average particle sizes of approximately 20 to 70 nm [26,27,28]. Due to its known activity, in this work *E. globulus* extract was used as a reference, considering that the potential of *G. officinalis*, *B. globosa* and *A. chilensis* extract in the preparation of ZnO NPs has not yet been explored. 

It is clear that different plant species contain varying types and concentrations of bioactive substances, which influence their antioxidant activity and has significant implications for their reducing ability as well as the synthesis of ZnO NPs. In this context, the main objective of our work was to investigate the effect of the AA of plant extracts on the physicochemical characteristics of ZnO NPs (production yield, chemical composition, particle size, PDI and ζ-potential). In order to accomplish this objective, four plant extract with different AA were used. 

## 2. Materials and Methods

### 2.1. Preparation of Plant Extracts

In the current investigation, ZnO NPs were produced using aqueous leaf extract from four different plant species, *G. officinalis*, *B. globosa*, *E. globulus*, and *A. chilensis*. Plant leaf extracts were prepared according to Solabomi et al. [14] with some modifications. Sufficient amounts of the fresh green leaves of all plants were collected from the garden of the Universidad de La Frontera in Temuco, Araucania Region, Chile (38°44′44.3″ S 72°37′01.8″ W), in May 2021 (austral winter season). The leaves were washed several times with distilled water, dried at 35 °C for three days, and cut into small pieces. A total of 10 g of the cleaned leaves was added to 200 mL of distilled water and heated at 60 °C for one hour. The resulting extract was filtered through Whatman number-1 filter paper, and the filtrate was collected in a closed vessel and stored at 4 °C for further use.

### 2.2. Qualitative Phytochemical Analysis

In order to identify the phytochemicals responsible for nanoparticles synthesis, the leaf extracts were subjected to plant-specific tests for the presence of tannins [29,30], saponins [31], flavonoids [29], terpenoids [31,32], quinones [33], coumarins [34] and glycosides [29,35]. These various standard procedures were performed following previously reported assays.

### 2.3. Quantitative Determination of Phytochemical Constituents

#### 2.3.1. Total Phenolic Content (TPC)

The Folin–Ciocalteu colorimetric method [31] was used to determine the total phenolic content of the four plant extracts. Briefly, the plant extract was diluted with distilled water to a known concentration. A total of 0.2 mL of diluted extract was mixed with 1 mL of Folin–Ciocalteu reagent (diluted 1:10 with distilled water) and 0.8 mL of 7.5% *w*/*v* sodium carbonate solution (Na_2_CO_3_). The mixture was shaken vigorously and incubated for 40 min at 45 °C for a complete reaction. The resulting absorbance was measured using a UV-Vis spectrophotometer (Thermo Scientific Evolution^TM^ 60S, Waltham, MA, USA) at 765 nm. Similarly, the calibration curve was constructed using gallic acid as the standard (20–100 mg/L). The results are expressed as mg gallic acid equivalents (GAE) per gram of dried weight (DW) of plant material (mg GAE/g DW) for three replicate analyses.

#### 2.3.2. Total Flavonoids Content (TFC)

The aluminium chloride (AlCl_3_) colorimetric method was used with some modifications for the flavonoid determination of the various plant extracts [36]. A total of 1 mL of diluted extract was mixed with 0.3 mL of 5% sodium nitrite (NaNO_2_) solution and 0.3 mL of 10% alcoholic aluminium chloride solution. The mixture was shaken and allowed to stand for 5 min at room temperature. Then, 2 mL of 1 M NaOH solution was added, and the absorbance was measured at 510 nm with a UV–Vis spectrophotometer Thermo Scientific Evolution^TM^ 60S. Similarly, the calibration curve was constructed using quercetin as the standard (40–200 mg/L). The results are expressed as mg quercetin equivalents (QE) per gram of dried weight (DW) of plant material (mg QE/g DW) and ± SD (standard deviation) for three replicate analyses.

#### 2.3.3. HPLC-DAD Analysis of Phenolic Compounds

The aqueous extracts obtained from the leaves were filtered through a 0.22 mm membrane and were analyzed by HPLC. Twenty microliters of each sample were injected into a Shimadzu HPLC (Model LC-20A Prominence, Kyoto, Japan) equipped with a C-18 column (250 mm × 4.6 mm ID; 5 μm particle size) maintained at 40 °C. The analysis was performed using a linear solvent gradient consisting of water (A) and methanol (B) as follows: 0–30 min, 90% A/10% B; 30–60 min, 60% A/40% B; 60–70 min, 55% A/45% B; 70–80 min and 90% A/10% B, at a flow rate of 1 mL/min. Phenolic compounds were monitored at 280 nm; UV spectra from 190 at 700 nm were used for peak characterization. The identification of phenolic compounds was based on the peak retention time compared to that of a reference standard.

#### 2.3.4. Ferric-Reducing Antioxidant Power (FRAP) Assay

The reducing power was determined according to the method described by Wong et al. [37]. First, the FRAP reagent was prepared from acetate buffer (pH 3.4), 10 mmol 2,4,6-trypyridyl-s-triazine (TPTZ) solution in 40 mmol HCl and 20 mmol FeCl_3_·6H_2_O solution in proportions of 10:1:1 (*v*/*v*), respectively. Then, 50 μL of the diluted extract was mixed with 3 mL of FRAP reagent, and after 20 min of incubation at 35 °C, absorption was measured at 593 nm using a UV-Vis spectrophotometer. The standard curve was constructed using iron (II) sulfate solution (100–2000 μM), and the results are expressed as μM Fe^2+^ per gram of dried weight (DW) of plant material (μM Fe^2+^/g DW) for three replicate analyses.

### 2.4. Synthesis of ZnO Nanoparticles

#### 2.4.1. Synthesis of ZnO NPs Mediated by Different Leaf Extracts

ZnO NPs synthesis was adopted from Rehana et al. [13]. First, 25 mL of 0.1 M zinc acetate dihydrate (Zn(CH_3_COO)_2_·2H_2_O) was added dropwise to 50 mL of plant extract under mechanical stirring and heated at 60 °C for 1 h. The color changes confirmed the formation of zinc hydroxide complexes. The dark brown precipitate was later separated by centrifuge at 10,000 rpm for 10 min and washed several times with distilled water until the pH of the suspension reached ~6–7. The solid product was transferred to a ceramic crucible, dried thoroughly, calcined at 400 °C for 3 h, and stored for further use. In order to verify the participation of phenolic compounds in nanoparticle production, quantitative changes in TPC during synthesis were monitored by the previously described Folin–Ciocalteu method. The effect of each aqueous leaf extract on the yield of ZnO NPs yield was investigated.

#### 2.4.2. Synthesis of ZnO NPs by Chemical Sol-Gel Method

**Figure 1:**
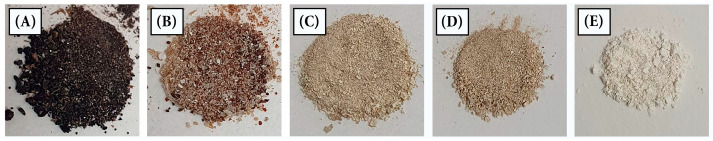
Appearance of ZnO NPs synthesized by green and chemical method. (**A**) *G. officinalis*, (**B**) *B. globosa*, (**C**) *E. globulus*, (**D**) *A. chilensis* and (**E**) NaOH.

ZnO NPs were also synthesized by traditional chemical methods adopted from Koutu et al. [38] to compare and evaluate the characteristics of the synthesized green nanoparticles. First, 50 mL of 0.1 M Zn(CH_3_COO)_2_·2H_2_O was added dropwise to 50 mL of 0.1 M NaOH under mechanical stirring and heated at 90 °C for 2 h under reflux. The color changes confirmed the formation of zinc hydroxide. The resulting white precipitate was later separated by centrifuge at 10,000 rpm for 10 min and washed several times with distilled water until the pH of suspension reached ~6–7. The solid product was transferred to a ceramic crucible, dried thoroughly, calcined at 400 °C for 3 h, and finally stored for further use. The appearances of the ZnO NPs powder synthetized using the green and chemical route are shown in Figure 1.

### 2.5. Characterization of ZnO NPs

The nanoparticles synthesized were characterized by their polydispersity index (PDI) and surface charge (ζ-potential) using a Zetasizer Nano ZS90 (Malvern Instruments, Inc., Malvern, UK). Optical properties were analyzed by UV-visible absorption spectroscopy (Thermo Scientific EvolutionTM 60S) in the range of 200–800 nm. Fourier-transform infrared spectroscopy (FTIR) and thermogravimetric analysis (TGA) were used to verify the involvement of the phenolic compounds in nanoparticle formation and to determine the percentage purity of the synthesized nanoparticles. Briefly, the powder samples were placed on top of the attenuated total reflection (ATR) crystal, and spectra were collected in the range of 400–4000 cm^−1^ using a Cary 630 FTIR instrument from Agilent Technologies. The TGA of the samples was obtained using a Perkin-Elmer STA 6000 by heating under atmospheric conditions from 25 °C up to 600 °C at 15 °C/min. The crystalline structure and constitution of the material were examined using a Smartlab Rigaku X-ray diffractometer, with a theta/theta (Bragg-Brentano) goniometer and D/teX Ultra 250 solid-state detector (Rigaku Corporation, Japan). XRD patterns were collected with Cu-Kα radiation (K = 1.5406 Å) at 30 kV and 40 mA, with Ni-filtered, in the range of 3–60° 2Theta, counting 0.5°/s per step of 0.01°. The morphology and particle size distribution of nanoparticles were determined by transmission electron microscopy (TEM) using a Carl Zeiss LIBRA^®^ 120 PLUS microscope. To estimate the average particle size and particle size distribution, approximately 100 NPs were measured in random fields of TEM images.

### 2.6. Data Analysis

The results were expressed as the mean ± standard deviation (SD) of at least three independent experiments. One-way ANOVA was used to analyze level of statistical significance between groups. Pearson’s correlation coefficient was determined to establish the relationship between different parameters. The statistical significance level was set to 0.05. All statistical analyses were conducted using the JMP statistical software.

## 3. Results and Discussion

### 3.1. Phytochemical Analysis of Plant Extracts

It is known that plants possess rich variability of active compounds, which can be used for the green synthesis of metal or metal oxide nanoparticles [39,40]. Hence, thoroughly studying the phytochemical profile of crude aqueous leaf extracts from *G. officinalis*, *B. globosa*, *A. chilensis*, and *E. globulus* is essential. Qualitative tests were conducted to identify the secondary metabolites in the extracts. The results obtained showed that the main constituents of the studied plant extracts were tannins, flavonoids, saponins, and terpenoids (Table 1). *G. officinalis* extract also contained cardiac glycosides, while *B. globosa* contained quinones, anthraquinone, and cardiac glycosides. On the other hand, the aqueous extract of *E. globulus* also included bioactive chemicals such as quinones and coumarins, while *A. chilensis* contained coumarins.

**Figure 2:**
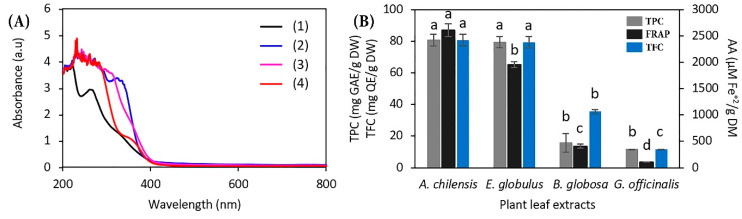
(**A**) UV-Vis spectrum of aqueous extracts: (1) *G. officinalis*, (2) *B. globosa*, (3) *E. globulus* and (4) *A. chilensis*. (**B**) Comparison of TPC, TFC, and AA of the different plant extracts. The same letters above the bars represent statistical non significance (*p* < 0.05).

The presence of chromophoric groups and aromatic rings in plant extracts was confirmed by UV–Vis spectrophotometry (Figure 2A). All spectra evidenced absorption bands in the ranges of 240–280 nm and 340–380 nm [31,41]. According to previous studies, bands ranging from 250 to 300 nm are characteristic of flavonoid derivatives, while bands ranging from 300 to 400 nm are characteristic of phenolic derivatives. It is known that the intensity of the absorption bands is directly proportional to concentration, suggesting that the *E. globulus* and *A. chilensis* extracts have a higher amount of phytochemical species compared with *B. globosa* and *G. officinalis*. In addition, the UV–Vis results reveal that the extracts showed variations in their chemical composition. 

Considering the vital role of phenolic compounds in the biogenic production of nanoparticles, the TPC and TFC of the different extracts were estimated. Additionally, antioxidant potential was investigated by the FRAP. The results obtained are presented in Figure 2B. 

**Figure 3:**
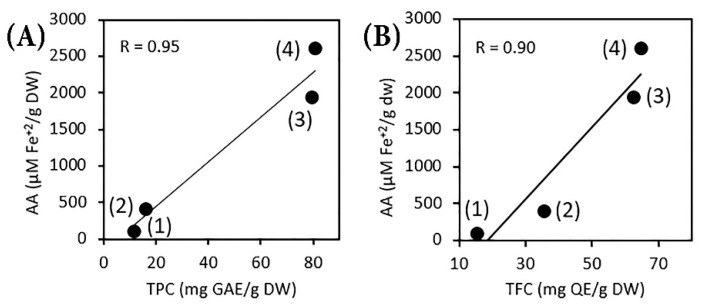
(**A**) Correlation between TPC and AA and (**B**) TFC and AA of (1) *G. officinalis*, (2) *B. globosa*, (3) *E. globulus* and (4) *A. chilensis*.

The TPC and TFC results show significant differences among the four extracts. *E. globulus* and *A. chilensis* leaf aqueous extract contained the highest amount of phenolic and flavonoid compounds compared with *B. globosa* and *G. officinalis*. With regard to the AA of the prepared extracts, *A. chilensis* extract showed the highest and most significant (*p* < 0.0001) AA, followed by *E. globulus*, *B. globosa*, and *G. officinalis*. These parameters (TPC, TFC and AA) exhibited a positive correlation (Figure 3A,B) between AA and TPC (R = 0.95) and between AA and TFC (R = 0.90), which implies that the AA of plant extracts varies proportionally with change in TPC and TFC. These results suggest that phenolic compounds are major contributors to the antioxidant activity of plant extracts.

**Figure 4:**
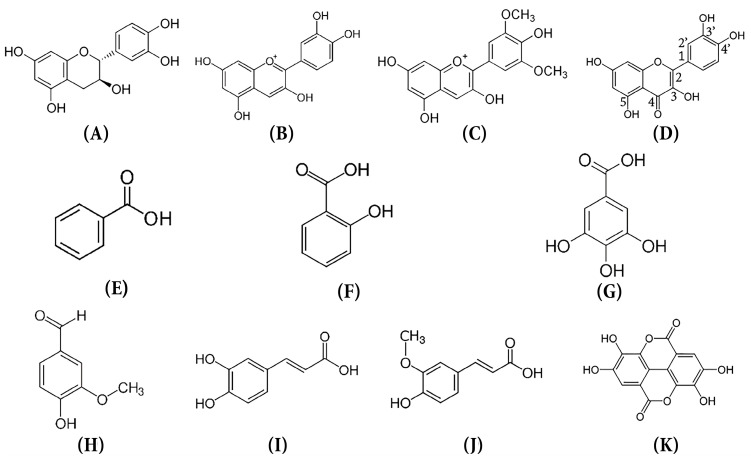
Chemical structure of standard phenolic compounds used in HPLC-DAD analysis. Assignments: (**A**) Catechin; (**B**) cyanidin; (**C**) malvidin; (**D**) quercetin; (**E**) benzoic acid; (**F**) salicylic acid; (**G**) gallic acid; (**H**) vanillin; (**I**) caffeic acid; (**J**) ferulic acid and (**K**) Ellagic acid.

Figure 2B shows that although *A. chilensis* and *E. globulus* extracts demostrated non-significant variability in TPC, their antioxidant capacity was quite different. This could be attributed to the different chemical structures of compounds in crude plant extracts. In order to obtain more information about the chemical nature of phenolic compounds, HPLC-DAD analyses were performed. A total of 11 phenolic compounds, –catechin, cyanidin, malvidin, quercetin, benzoic acid, salicylic acid, gallic acid, vanillin, caffeic acid, ferulic acid, and ellagic acid– were used in this study. Figure 4 and Table 2 show the quantification and chemical structure of the phenolic compounds, respectively.

Phenolic compounds comprise one (phenolic acids) or more (polyphenols) aromatic rings containing one or more hydroxyl substituents [42,43]. Indeed, it is known that the number and arrangement of the phenolic hydroxyl groups and the degree of hydroxylation or extent of alkylation and glycosylation within each group cause the difference in the antioxidant activities of phenolic compounds [42,44]. In this context, gallic acid (Figure 4G) with three hydroxyl groups exhibits better antioxidant properties than salicylic acid (Figure 4F), with only one hydroxyl group bound to the aromatic ring. Similarly, caffeic acid (Figure 4I) with two hydroxyl groups exhibits better antioxidant properties than ferulic acid (Figure 4J) and vanillic acid (Figure 4H), which have only one hydroxyl group bound to the aromatic ring. At the same time, ferulic acid has better antioxidant properties than vanillic acid due to the presence of an –CH=CH–COOH group in this structure, which enhances the antioxidant activity more than the –COH group [44,45].

As for flavonoids, the data presented in the literature suggests that quercetin (Figure 4D) is a more effective antioxidant than catechin (Figure 4A). Although catechin has the same number and location of hydroxyl substituents as quercetin, the conjugation of the C_2_–C_3_ double bond with the C_4_ carbonyl group and C_3_ and C_5_ hydroxyl groups (Figure 4D) of the quercetin molecule contribute to radical scavenging, metal chelating and the stabilization of the phenoxyl radical produced by hydrogen donation [44,45].

According to Angelo et al. [46], the antioxidant activity of structurally diverse phenolic compounds in *decreasing order* was ellagic acid > quercetin > catechin > caffeic acid > cyanidin > ferulic acid > vanillic acid. Similar results were reported by Bhutto et al. [42], who observed that the *decreasing order* of antioxidant capacity values was quercetin > caffeic acid > ferulic acid > salicylic acid; however, benzoic acid did not exhibit antioxidant activity. Hence, the results shown in Table 2 allow us to conclude that the highest concentration and highest AA of compounds such as catechin, quercetin, and caffeic acid contribute significantly to the antioxidant capacities of *E. globulus* and *A. chilensis* extracts. By contrast, *B. globosa* and *G. officinalis* extracts showed the lowest antioxidant activity due to the low concentration and inferior antioxidant properties of malvidin and salicylic acid.

### 3.2. Characterization of ZnO NPs

#### 3.2.1. UV–Visible Spectroscopy

**Figure 5:**
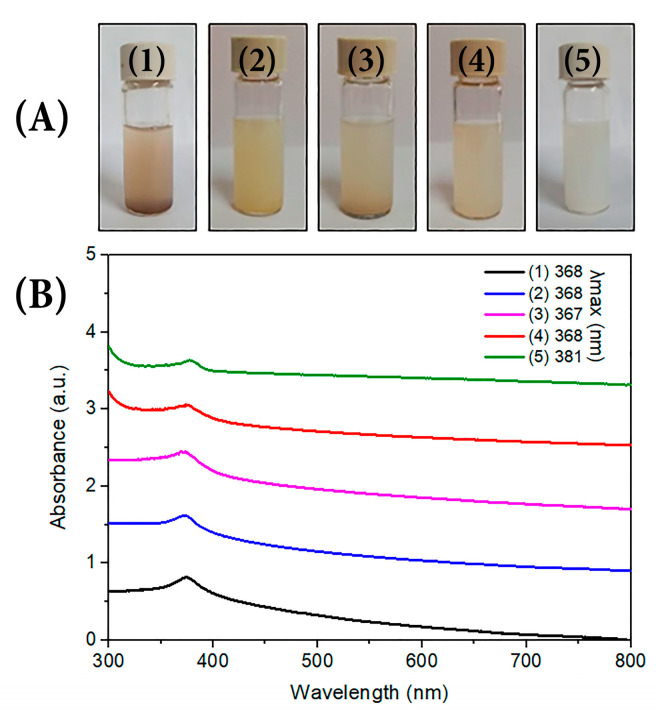
(**A**) Photographs of ZnO NPs dispersion in water. (**B**) UV–vis spectra of ZnO NPs synthesized by biological and chemical methods. (1) *G. officinalis*, (2) *B. globosa*, (3) *E. globulus*, (4) *A. chilensis* and (5) NaOH.

ZnO NPs were synthesized using aqueous leaf extract from four different plant species with diverse antioxidant activities. Visual examination confirmed the formation of ZnO NPs. The color of the reaction mixture changed from yellow to pale brown during the reaction and from light to dark brown at 400 °C using a muffle furnace for 3 h. The solids obtained were dispersed in distilled water by ultrasound for 5 min (Figure 5A), and their optical absorption was recorded using a UV–vis spectrophotometer. The UV absorption spectra are shown in Figure 5B. All of the samples exhibited strong absorption peaks centered at 368, 367, 368, and 368 nm for *A. chilensis*, *E. globulus*, *B. globosa*, and *G. officinalis*, respectively, which are characteristic peak for ZnO [47,48,49]. Green synthesized nanoparticles showed a higher degree of blue shift compared with chemically synthesized nanoparticles (approx. 381 nm). This suggests that the particle size is larger for the chemical method compared with that of prepared samples using plant extracts [7,47,50]. 

Due to importance of ZnO semiconductor as an opto-electronic material [51], its band gap was calculated based on the equation Eg(eV) = (hc/k) = 1240/k (nm), where Eg is the optical band gap, h is Planck’s constant, c is the speed of light and k is the wavelength of maximum absorption. The calculated optical band gap values are 3.36, 3.37, 3.36, 3.36, and 3.24 eV for *A. chilensis*, *E. globulus*, *B. globosa*, *G. officinalis*, and chemical method, respectively. These values are in good agreement with those reported in earlier studies [16,52,53].

#### 3.2.2. XRD Analysis

**Figure 6:**
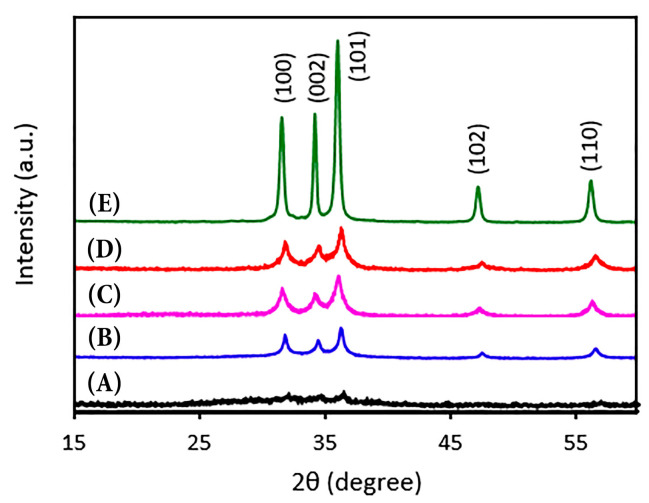
XRD patterns of ZnO NPs synthesized by biological and chemical methods. (**A**) *G. officinalis*, (**B**) *B. globosa*, (**C**) *E. globulus*, (**D**) *A. chilensis* and (**E**) NaOH.

The XRD patterns of the samples synthesized by the chemical and green routes are shown in Figure 6. All of the diffraction patterns were identified as ZnO with a hexagonal wurtzite structure in accordance with the Crystallography Open Database, COD ID 2300113. In Figure 6, it can also be seen that the diffraction peak intensity decreases with the use of different plant extracts, suggesting that the degree of crystallinity of samples decreased due to the anomalous atomic order inside nanoparticles. The presence of small and broad peaks on the diffractogram could be also attributed to crystallite size reductions.

Based on the pattern profile analysis, the crystallite size (D) was estimated by Debye–Scherrer as follows [54,55,56]:

(1) $$D=\frac{k\lambda }{\beta_{\left(hkl\right)}\cos\theta }$$

where λ is the wavelength of the X-ray radiation (Cu−Kα = 0.1541 nm), k is known as the Scherrer constant, which is for a spherical crystal 0.94, β is the full line width at the half-maximum (FWHM) of the hkl intensity peak, and θ is the Bragg angle. These results are supported by the Williamson–Hall plot (W–H plot) using the uniform deformation model (UDM) (Equation (2)), which considers a crystal to be isotropic and, consequently, its properties to be independent of the crystallographic direction along which the measurement is taken [55].

(2) $$\beta_{\left(hkl\right)}\cos\theta =\frac{k\lambda }{D}+4\varepsilon sin\theta$$

The linear regression of the data obtained between βcosθ vs. 4sinθ indicates the crystallite size and lattice strain (ε) through their intercept and slope, respectively. The estimated values of crystallite size and lattice strain are presented in Table 3. Crystallite sizes of green-prepared particles were found in a range from 2.2 to 17.78 nm. By contrast, ZnO NPs prepared by the chemical method showed a crystallite size between 22.8 and 29.5 nm. The lower values for biogenic nanoparticles can be attributed to the efficient role of polyphenolic compounds derived from plants as capping and stabilizing agents during synthesis. For ZnO NPs prepared using *G. officinalis* it was not possible to estimate the crystallite size due to the presence of amorphous carbon on the particles which masked the ZnO. This low intensity generated by the ZnO signals made it difficult to identify the individual peaks.

On the other hand, the lattice strain values estimated using the W–H plot decreased with increasing crystalline domain size. Lattice strain measures the distribution of the lattice constant arising from crystal imperfections such as lattice inhomogeneities. The larger the strain, the greater the concentration of defects and imperfections [55,57]. Thus, the presence of phenolic compounds limited particle growth, inhibited crystallization and promoted atomic and lattice disorder, which could affect the reactivity of ZnO NPs [58,59].

#### 3.2.3. FT-IR Analysis

**Figure 7:**
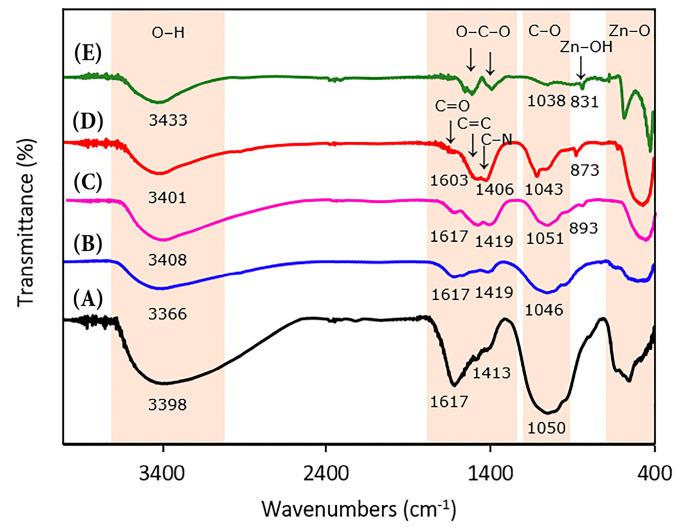
FTIR spectra of ZnO NPs prepared using (**A**) *G. officinalis*, (**B**) *B. globose*, (**C**) *E. globulus*, (**D**) *A. chilensis* and (**E**) NaOH.

Figure 7 shows the FTIR absorption spectra of synthesized ZnO NPs. All of the spectra demonstrated a broad absorption peak located at approximately 3400 cm^−1^ corresponding to H–O vibration, in addition to the characteristic bands of Zn–O stretching from 400 cm^−1^ to 600 cm^−1^, which confirmed the presence of ZnO. Nanoparticles prepared by chemical method showed absorption peaks at 1038, 1383, and 1507 cm^−1^ corresponding to C–O stretching, symmetric O–C–O stretching, and asymmetric O–C–O stretching vibration, respectively, denoting the presence of acetate groups from the precursor salt used in the synthesis. On the other hand, the bands observed at 1620, 1480, 1400, and 1050 cm^−1^ for ZnO NPs synthesized using aqueous plant extract correspond to the stretching vibration of C=O, C=C, C–N, and C–O [28,49,60] due to the presence of residual organic extract that remained on the particles after calcination, which caused changes in the physical appearance of all the powders obtained, varying in color from light to dark brown or black (see Figure 5B). The use of *G. officinalis* to produce ZnO NPs caused an increase in the peak intensity in FTIR (Figure 7A), suggesting that the purity of the sample decreased due to a higher content of residual phenolic compounds. The FTIR results indicate that the phenolic compounds in the plant extracts were successfully incorporated into the surface of nanoparticles during the synthesis.

#### 3.2.4. TGA Analysis

**Figure 8:**
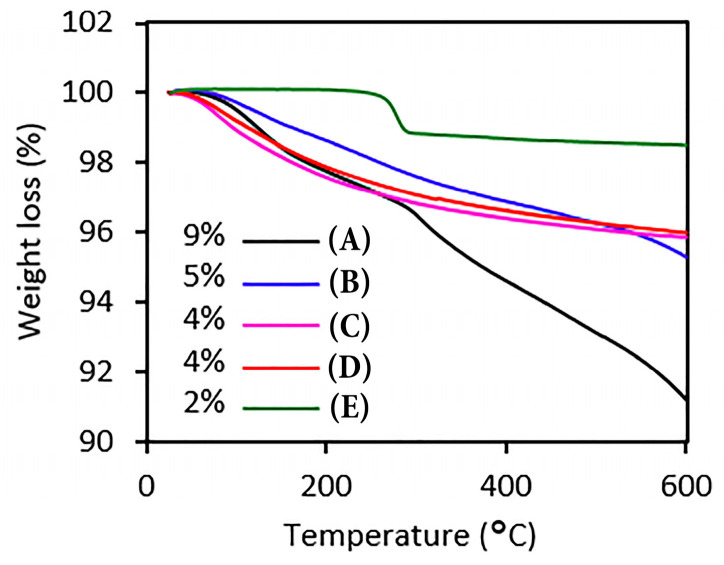
TGA analysis of ZnO NPs synthesized using (**A**) *G. officinalis*, (**B**) *B. globose*, (**C**) *E. globulus*, (**D**) *A. chilensis* and (**E**) NaOH.

To determine the thermal stability and weight percentages of the bonded plant phenolic compounds over the ZnO NPs prepared by the green synthesis method, a TGA analysis was performed (Figure 8). ZnO NPs obtained by the chemical method were also studied using TGA. Chemical nanoparticles showed a weight loss of 2% due to the evaporation of water molecules. In comparison, samples produced by plant extracts exhibited continuous weight losses ranging from 9% to 4%, which are related to the thermal decomposition of water and bioactive compounds in plant extracts bonded to the nanoparticle surfaces. TGA analysis indicates that calcination temperatures higher than 400 °C are required to remove the organic compounds on the surface of ZnO NPs. The highest weight percentage of organic matter was evidenced when the *G. officinalis* extract was used in the synthesis of ZnO NPs. The results in Figure 5d are in good agreement with the FTIR spectra. 

#### 3.2.5. DLS Analysis

The PDI and ζ-potential of ZnO NPs suspended in distilled water were also evaluated. Samples produced using *A. chilensis*, *E. globulus*, *B. globosa*, and *G. officinalis* exhibited PDI values of 0.2, 0.2, 0.3 and 0.5, respectively, while the sample synthetized by the chemical method showed a PDI value of 0.1. Typically, PDI values for ZnO NPs between 0.1 and 0.3 indicate a narrow size distribution, whereas a PDI > 0.5 indicates that the sample has a very broad size distribution [61,62,63]. In this sense, PDI measurements suggested that ZnO NPs with monodispersed size distributions were obtained, except when *G. officinalis* extract was used. In this case, a high PDI indicated some heterogeneity in the particle-sizes distribution.

Next, the obtained average ζ-potential value was positive for chemically prepared ZnO NPs (18.8 mV). By contrast, ZnO NPs synthesized using aqueous plant extract showed negative zeta potential (−14.5, −14.0, −16.0 and −16.1 mV for *A. chilensis*, *E. globulus*, *B. globosa*, and *G. officinalis*, respectively) due to the surface coating with negatively charged organic compounds. The ζ-potential defines the stability of particle dispersions and is a typical measurement of the surface charge on a particle. Ζ-potential values higher than +30 mV or lower than −30 mV are indicative of stable nanoparticle dispersions [64]. Therefore, the ζ-potential values of the prepared ZnO NPs prepared indicates a weak repulsive force between the particles, which promotes their aggregation.

#### 3.2.6. TEM Analysis

**Figure 9:**
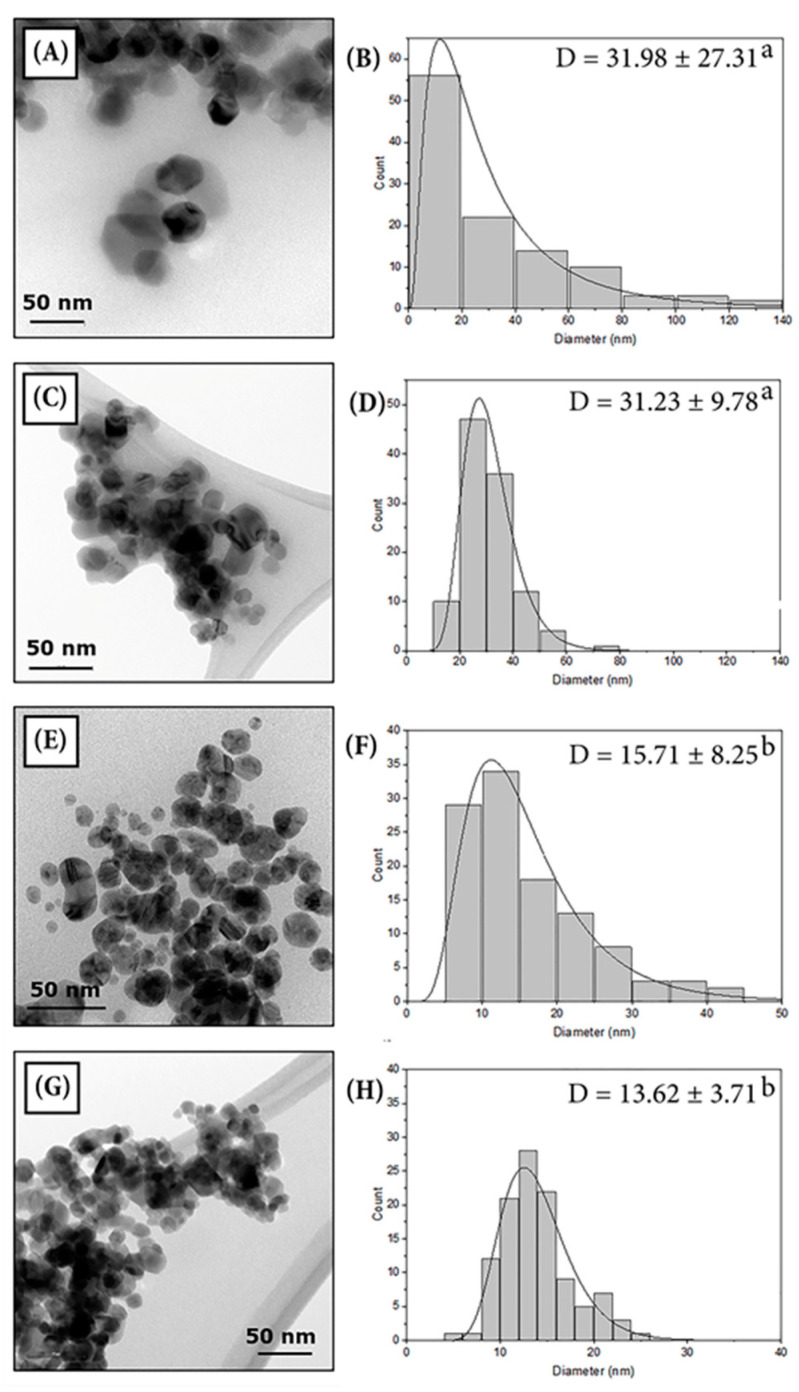
TEM images and size distribution of ZnO NPs prepared from: (**A**,**B**) *G. officinalis*, (**C**,**D**) *B. globosa*, (**E**,**F**) *E. globulus*, and (**G**,**H**) *A. chilensis*. The results are expressed as mean diameter ± standard deviation. The same letters next to the average diameter values represent statistical non significance (*p* < 0.05).

The particle size distribution and morphology of ZnO NPs prepared from different plant extracts were explored using TEM (Figure 9). The TEM images showed the formation of predominantly spherical-shaped ZnO NPs. All size distribution patterns fit best to a log-normal distribution with an average diameter (D) of 31.98 ± 27.31, 31.23 ± 9.78, 15.71 ± 8.25, and 13.62 ± 3.71 nm using extract of *G. officinalis*, *B. globosa*, *E. globulus*, and *A. chilensis,* respectively. The particle size distribution obtained by TEM was higher compared with the crystallite sizes calculated from the XRD data, because a crystallite is a single crystal with a long-range order of atoms. However, a particle often has more than one crystallite.

Different plant extracts have a clear effect on particle size and distribution. The use of *G. officinalis* extracts exhibited the highest average diameter value and standard deviations, suggesting non-uniform particle size distribution, probably caused by the presence of the larger amount of residual amorphous carbon residual on the ZnO (Figure 9A), formed by the incomplete combustion of organic extract, which can severely affect the properties of ZnO NPs. It is known that, during the synthesis of ZnO NPs, phenolic compounds act as capping agents to stabilize the nanoparticles, preventing further growth and agglomeration. However, they are often detrimental to the construction of functionalized nanomaterial, especially those that require clean inorganic interfaces and surfaces [65,66]. Therefore, during synthesis, a calcination step is required to obtain the ZnO phase and remove organic ligands from plant extracts. In this work, it was shown that the temperature employed in thermal treatment did not remove all organic matter from the produced nanoparticles. Hence, the calcination of powders at 400 °C converted the phenolic compounds surrounding the ZnO NPs into amorphous carbon, which caused an increase in the average particle size as a consequence of agglomeration and particle coarsening.

**Figure 10:**
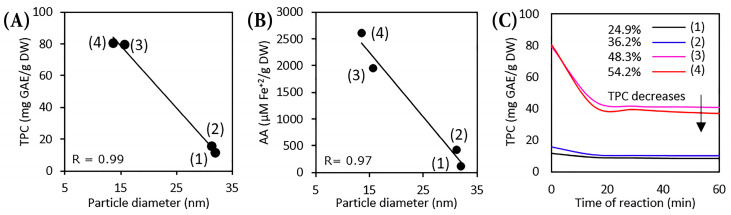
Correlation between particle diameter and: (**A**) TPC and (**B**) AA. (**C**) Change in the TPC of the plant extracts used to synthesize ZnO. (1) *G. officinalis*, (2) *B. globose*, (3) *E. globulus*, and (4) *A. chilensis*.

Particle diameter showed a negative correlation with TPC and AA (Figure 10A,B). To put it another way, plants containing higher TPC and AA made it possible to obtain nanopowders with smaller particle size and a narrow particle size distribution compared with plants with a lower TPC and lower AA, which can be attributed to the amount of phenolic compounds involved in the process being greater during synthesis with *A. chilensis* and *E. globulus*. This was confirmed through monitoring the TPC during the synthesis reaction (Figure 10C). In this regard, a remarkable reduction in TPC was noted as synthesis progressed: 54.2, 48.3, 36.2, and 24.9% when *A. chilensis*, *E. globulus*, *B. globosa*, and *G. officinalis*, were used, respectively. Therefore, not all phenolic compounds present in plant extracts act as capping and stabilizing agents. Only phenolic compounds with high antioxidant activity actively participate in the reaction leading to the formation of nanoparticles. The results of Figure 9 and Figure 10 allows to confirm the capping and stabilizing effect of the phenolic compounds from plant extracts on the surface of the ZnO NPs.

### 3.3. Effect of the Antioxidant Activity of Plant Extracts on the Yield of ZnO NPs

**Figure 11:**
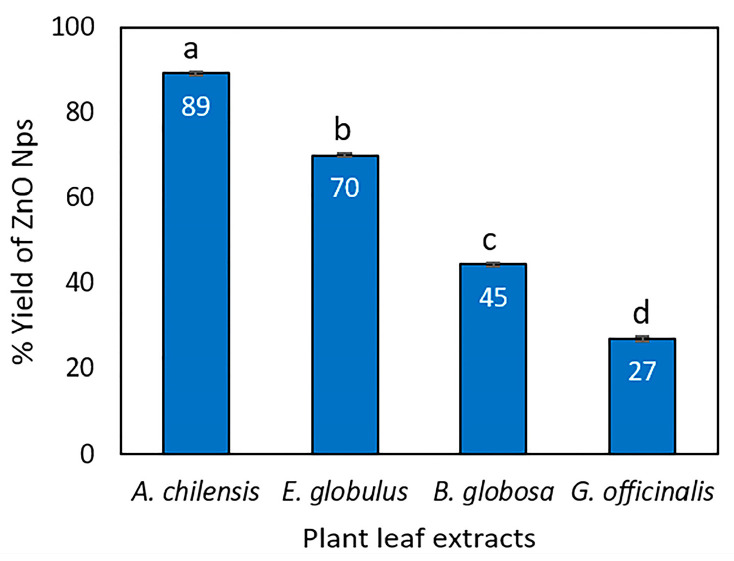
Effect of different plant extracts on the of ZnO NPs. The same letters above the bars represent statistical non-significance (*p* < 0.05).

The effect of the AA of the four plant extracts on the yield of ZnO NPs was investigated. Figure 11 summarizes the yields of the synthetized ZnO NPs. The results obtained indicate that the yield of ZnO NPs varied according to the plant species used in the preparation of the extract. The highest yield was achieved with *A. chilensis* extract, while *G. officinalis* extract had the lowest. It was demonstrated that plant extracts containing higher TPC values show high AA. Similarly, plant extracts with high AA evidenced the best chelating and reducing abilities in the synthesis of ZnO, reaching a maximum yield of 89.37%. Despite the fact that several authors have reported the use of *E. globulus* extract in the synthesis of ZnO NPs, none of these earlier studies included the yield of nanoparticles obtained. 

**Figure 12:**
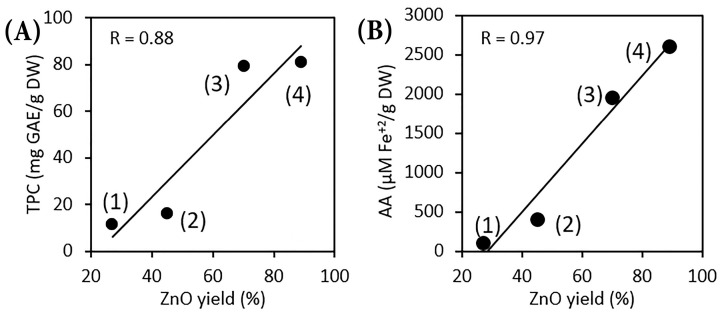
Correlation between (**A**) TPC and yield of ZnO NPs and (**B**) AA and yield of ZnO NPs of (1) *G. officinalis*, (2) *B. globosa*, (3) *E. globulus*, and (4) *A. chilensis*.

The yield of ZnO NPs was significantly correlated with the values of TPC (R = 0.88) and AA (R = 0.90) of the plant extracts (Figure 12), indicating that phenolic compounds are the major contributors to the formation of ZnO NPs. The mechanism and the biomolecules responsible for nanoparticle formation via plant extracts have not yet been fully documented. Barzinjy and Azeez [67] reported a plausible mechanism for the formation of ZnO NPs formation using *E. globulus* leaf extracts, which is described as:

stirring

nZn^2+^ + 2Ar-(OH) → nZn^0^ + 2nAr = O + 2nH^+^ (3)

stirring

2(x+1)Zn^0^ + 2Ar = O + yO_2_ → 2(Zn^0^-Zn_x_O_y_-Ar = O) (4)

Several reports indicate that both the hydroxyl and the carboxylic acid groups of phenolic compounds in plant extract are responsible for the formation of ZnO NPs. In particular, the hydroxyl groups are responsible for the reduction of Zn^2+^ ions to Zn^0^, while the carboxyl groups act as stabilizing agents [68,69,70,71]. This might cause the Zn^0^-phenolate complex through chelating effect, bringing about nucleation, growth, and the capping of nanoparticles at 60 °C [5,72]. Subsequently, this complex undergoes direct decomposition at high temperatures (≥400 °C) and leads to ZnO NPs [67].

The results obtained in this work suggest that it is possible to use antioxidant activity as an indicator of the reducing capacity of the plant extracts; in this way, predictions can be made about the potential of plant extracts with regard to the formation and production of nanoparticles.

### 3.4. Green Synthesis of ZnO NPs: Challenges

There are still many challenges that need to be overcome in order to replace conventional chemical methods of nanoparticle synthesis with biogenic ones. The results obtained in this study show an improvement in the physicochemical characteristics and yield of ZnO NPs when a plant extract with high TPC and high AA was used. The type of plant and the calcination temperature used for the fabrication of nanoparticles influenced the residual organic matter content in the final powder. In this sense, the evaluation of ZnO purity and its effect on nanoparticle reactivity must be addressed. Additionally, the achieved yield of the materials synthesized via the green route was near the yield obtained by the chemical method (97.61%). Considering that the precursor salt of zinc (in our case, Zn(CH_3_COO)_2_.2H_2_O) represented 70% of the cost of nanoparticle production, reaction yield is one of the most important factors to consider in determining the overall cost required for its preparation. Although a large number of researchers have documented the biogenic synthesis of ZnO, only a few studies have reported on the yields obtained. This lack of knowledge can limit the scalability of the route. 

It is known that phenolic profiles, as well as antioxidant activity in leaves, can change according to plant species, environmental conditions, geographic origin, and seasonal factors. Therefore, to guarantee the efficient biosynthesis of nanoparticles, it is crucial to know the temporal variation in plant extract production.

In this work, the nanoparticles were produced at atmospheric pressure and mild temperature to avoid the potential degradation of phenolic compounds. Compared to biological synthesis, the traditional method often requires an hours-long reaction time. Therefore, using green routes supposedly results in decrease in energy consumption (Table 4), contributing to reducing the environmental and economic impact of these processes. However, a life-cycle analysis is necessary to quantify the impact of nanoparticle production, from the materials used to make it to its application and the final layout, thereby reducing the environmental impacts even further.

## 4. Conclusions

The findings of our study show that the four plant extracts used for the synthesis of ZnO NPs have different antioxidant activities, due to the presence of various phytochemical species, such as catechin, malvidin, quercetin, caffeic acid, and ellagic acid. *A. chilensis* extract showed the highest TPC, TFC, and AA, followed by *E. globulus*, *B. globosa*, and *G. officinalis*. A positive linear correlation was found between TPC and AA, which suggests that phenolic compounds are significant contributors to the AA in plant extracts.

When the synthesis was carried out, it was found that the reaction physicochemical properties of ZnO NPs (such as yield production, chemical composition, particle size, PDI, and ζ-potential) vary depending on the plant species used. The contribution of polyphenolic compounds in the nanoparticle formation using plant extracts was confirmed through the reduction in TPC during the synthesis reaction. ZnO NPs synthesized using *A. chilensis* extract achieved the highest yield percentage and a smaller particle diameter, while the use of *G. officinalis* extracts had the lowest yield percentage and larger particle diameter. A high content of residual organic matter in the synthesized product using *G. officinalis* caused an increase in the heterogeneity of the particle distributions and particle size, which is often detrimental to the construction of functionalized nanomaterials and can also have repercussions on the reactivity of the sample. Hence, the aqueous extract with a higher AA was the best candidate for the formation of ZnO NPs. Our results provide a novel approach for predicting the efficiency of extracts in the formation of ZnO NPs.

## Figures and Tables

**Table 1:** Preliminary qualitative phytochemical analysis of the various aqueous plant extracts studied in this work.

Phytochemical Constituents	Plant Leaf Extracts			
	*G. officinalis*	*B. globosa*	*E. globulus*	*A. chilensis*
Tannins	+	+	+	+
Saponins	+	+	+	+
Flavonoids	+	+	+	+
Terpenoids	+	+	+	+
Quinones	-	+	+	-
Coumarins	-	-	+	+
Anthraquinone glycosides	-	+	-	-
Cardiac glycosides	+	+	-	-

- absence and + presence.

**Table 2:** Identification and quantification of phenolic compounds present in aqueous plant extracts by HPLC DAD.

Identification/Subclass		Concentration (mg/L)			
		*G. officinalis*	*B. globosa*	*E. globulus*	*A. chilensis*
	Flavonoids				
A	Catechin	2.6	-	361.8	-
B	Cyanidin	-	0.7	-	-
C	Malvidin	-	37.7	20.2	14.2
D	Quercetin	-	-	85.1	54.5
	Benzoic acids (-COOH substituted)				
E	Benzoic acid	7.0	3.1	-	-
F	Salicylic acid	42.5	4.6	0.5	-
G	Gallic acid	0.1	-	-	0.3
	Cinnamic acids (-OCH_3_ substituted)				
H	Caffeic acid	2.1	2.4	124.7	9.5
I	Ferulic acid	0.3	-	-	-
	Others				
J	Ellagic acid	-	-	5.1	9.2
K	Vanillin	1.2	0.5	4.7	-

**Table 3:** Crystallite size calculated using Debye-Scherrer and Williamson-Hall.

Plant Extracts	Crystallite Size XRD (nm)		ε × 10^−3^
	Scherrer’s Equation	W–H Plots	
*G. officinalis*	---	---	---
*B. globosa*	2.2	6.0	6.5
*E. globulus*	12.3	17.8	7.8
*A. chilensis*	8.0	4.5	6.9
Chemical method	22.8	29.5	0.7

**Table 4:** Energy consumption of each type of equipment used in this study for the green and chemical synthesis of ZnO NPs at laboratory scale.

Equipment		KW	Green Synthesis		Chemical Synthesis	
			Time (h)	KW/h	Time (h)	KW/h
Energy	Heating plate	0.45	1.0	0.45	4.0	1.80
	Centrifuge (13,000 rpm)	0.46	0.5	0.23	2.0	0.91
	Dryer	4.60	3.0	13.80	3.0	13.80
	Muffle	1.60	24.0	38.40	24.0	38.40
Total		7.11	28.5	52.88	33.0	54.99

## Data Availability

Not applicable.
